# Hospital Construction

**Published:** 1887-12-10

**Authors:** Albert Napper

**Affiliations:** Chichester Place, Guildford


**Conclusion.**

### THE EDITOR'S LETTER-BOX.

[Our Correspondents are reminded that prolixity is a great bar to publication, and that brevity of style and conciseness of statement greatly facilitate early publication.]

### Hospital Construction.

The very interesting discussion on the subject of Hospital Construction, as reported in The Hospital of 26th November, has been read by me with much interest. Some pertinent remarks were offered on many subjects, including some rather questionable opinions on the subject of draughts, but upon these I do not propose making any comment. The question that appeared to command the most interest, and about which every speaker seemed to be equally abroad, was that of ventilation, and it is to this subject that I shall confine my remarks. Many years' careful attention to and study of the subject have led me to the conclusion that the only scientific attempt at a solution of this problem is that of Tobin, which to a certain extent is efficacious, and my endeavour has been to complete what he has so well begun. His system is based upon gravitation, and properly so; but he fails to obtain the full benefit of his plan, from the fact of the air having to rise in his tubes before entering the chamber, by which much power of gravitation is lost. Again, he makes no effectual means of escape for the rarefied air, which is a point of the utmost importance. All attempts hitherto have been to provide an exit for it into the open air direct. To effect this is a physical impossibility, as no warm, rarefied air, can force its way out against cold, condensed air. Having arrived thus far in my theoretical problem, I had an opportunity, accidentally, afforded me, about two years since, of testing it in the roof of a large church, in which a zinc tube of about three feet in diameter, tapering off to about eighteen inches, was constructed to carry off the foul air into a chimney communicating with the open air. Of course, as I expected, there was no proof that any foul air ever made its exit by that source, but there was ample evidence that it was the medium by which a free supply of pure air was admitted. This shaft was in connexion with a large circular opening in the ceiling, protected by open fretwork, from which the gas chandelier was suspended. The zinc shaft occupied one-half of the opening, by which an ample supply of fresh air was admitted, but it was found necessary to allow the foul air to escape somewhere to render the ventilation complete. This was easily effected by the removal of some boards from the other half of the circle. The effect was very remarkable. Heated foul air came pouring up through this half, whilst cold pure atmosphere flowed down through the other, to the great relief of the congregation. I was enabled to place one hand in the cold downpower, whilst I held the other in the heated upflow. About a year after this a public hall was erected, in which I am interested, and I took the opportunity of getting it ventilated in accordance with the above method, and with the most satisfactory result. The hall extends partly up into the roof, leaving a space of about four feet between the ceiling and the ridge. A cupola on the roof, carried down through the ceiling, but not communicating with the chamber above it, freely admits the cold air into a small shallow chamber inside the ceiling of the hall, and at each end of which is a flap that is regulated by a string, so that any requisite quantity of air can be admitted. These flaps are not allowed to descend to quite a horizontal position, so that the air is canted upwards to the ceiling, thereby preventing any draught. The air thus admitted descends by its own gravity to the floor, and forces the warm, rarefied atmosphere upwards. As this cannot by any possibility escape through any opening into the external air, an arrangement is made that allows it to escape through the ceiling by a series of small trap-doors into the less resisting atmosphere of the roof, where it partly contracts and partly escapes into the open air, thus keeping up a circulation that nothing can put out of order. This is the first experiment that has been tried, and has not yet been tested, in a building of several rooms; but I can see no reason why it should not in this case be equally successful. The principle once established, the difficulties of detail will soon be overcome. I have had sufficient experience of the working of the above system to warrant me in strongly recommending its adoption. I shall be pleased to show the working of it to any gentleman who may favour me with a visit. Albert Napper.

Chichester Place, Guildford, December 1st.

